# Comparative effectiveness and safety of colistin-based versus high-dose ampicillin/sulbactam-based combination therapy for nosocomial pneumonia caused by carbapenem-resistant *Acinetobacter baumannii*

**DOI:** 10.1128/aac.01880-24

**Published:** 2025-04-23

**Authors:** Jaehoon Lee, Imchang Lee, Ki-Byung Lee, Seung Soon Lee

**Affiliations:** 1 Hallym University College of Medicine 96664, Chuncheon, Gangwon, South Korea; 2 Department of Life Science, Multidisciplinary Genome Institute, Hallym University 26727, Chuncheon, Gangwon, South Korea; 3 Division of Pulmonary, Allergy and Critical Care Medicine, Department of Internal Medicine, Hallym University Chuncheon Sacred Heart Hospital, Hallym University College of Medicine 96664, Chuncheon, Gangwon, South Korea; 4 Division of Infectious Diseases, Department of Internal Medicine, Hallym University Chuncheon Sacred Heart Hospital, Hallym University College of Medicine 96664, Chuncheon, Gangwon, South Korea; Johns Hopkins University School of Medicine, Baltimore, Maryland, USA

**Keywords:** carbapenem-resistant *Acinetobacter baumannii*, nosocomial pneumonia, colistin, sulbactam, treatment outcome

## Abstract

Differences exist between Infectious Diseases Society of America guidance and European Society of Clinical Microbiology and Infectious Diseases guidelines on treating pneumonia caused by carbapenem-resistant *Acinetobacter baumannii* (CRAB). This study compared the outcomes of colistin-based and high-dose ampicillin/sulbactam-based combination therapies in patients with CRAB nosocomial pneumonia. A retrospective cohort study was conducted at a university-affiliated hospital in South Korea. Patients received either a colistin-based regimen with a loading dose followed by a maintenance dose (June 2021–May 2022) or a high-dose ampicillin/sulbactam-based regimen with sulbactam 9 g/day (October 2022–September 2023). The primary outcome was 28-day all-cause mortality; secondary outcomes included 14-day/28-day clinical success rates and 14-day/28-day kidney injury based on the Risk, Injury, Failure, Loss, End-stage renal disease score. Logistic and Poisson regression analyses were used to compare outcomes between groups. Among 179 patients enrolled in the study, 84 received the colistin-based regimen and 95 received the high-dose ampicillin/sulbactam-based regimen. The ampicillin/sulbactam group showed significantly lower 28-day mortality (20% vs. 61%; adjusted relative risk [aRR] = 0.16, 95% CI 0.08–0.32). Clinical success rates were higher in the ampicillin/sulbactam group at both 14 days (39% vs. 23%; aRR = 2.19, 95% CI 1.10–4.37) and 28 days (55% vs. 32%; aRR = 2.71, 95% CI 1.14–5.20). Additionally, 28-day kidney injury was lower in the ampicillin/sulbactam group (0.63 ± 1.16 vs. 1.06 ± 1.35; aRR = 0.56, 95% CI 0.40–0.79). High-dose ampicillin/sulbactam-based combination therapy demonstrates superior outcomes over colistin-based combination therapy for CRAB nosocomial pneumonia, including lower mortality, higher clinical success rates, and reduced kidney injury.

## INTRODUCTION

Carbapenem-resistant *Acinetobacter baumannii* (CRAB) was classified as an urgent antibiotic resistance threat by the US CDC in 2019 and as a critical priority pathogen by the WHO in 2024 (1, 2). In South Korea, the carbapenem resistance rate of *A. baumannii* began to skyrocket between 2005 and 2010 due to the rise in extended-spectrum beta-lactamase-producing Enterobacteriaceae infections, coupled with increased carbapenem usage and insufficient infection control measures (3). As a result, carbapenem resistance rates in *A. baumannii* reached 90% in most hospitals (4). While infections caused by *A. baumannii* were initially not considered a major treatment target due to its low virulence, CRAB gradually developed resistance to most antibiotics and has become a common cause of infections in critically ill patients, particularly in intensive care units, where CRAB bacteremia and pneumonia are associated with poor prognosis (5, 6).

To date, none of the new antibiotics for CRAB pneumonia, such as cefiderocol and sulbactam-durlobactam, have been approved in South Korea (5, 7). Clinicians have been forced to rely on polymyxins to treat CRAB infections, with few other therapeutic options available. Colistin therapy, based on well-established pharmacokinetic/pharmacodynamic (PK/PD) data, has been widely used for treating CRAB infections in most institutions (6, 8, 9). The European Society of Clinical Microbiology and Infectious Diseases (ESCMID) treatment guidelines for moderate to severe CRAB infections suggest combination therapy with two *in vitro* active agents among the available antibiotics, favoring polymyxin- or high-dose tigecycline-based therapies (7). While colistin can be effective and safe when carefully dosed according to renal function, recent reports have shown unsatisfactory clinical outcomes, along with the major disadvantage of nephrotoxicity associated with the drug (5). Notably, colistin, a polymyxin E, has inferior PK/PD properties compared to polymyxin B, resulting in poor lung parenchymal concentration (5, 10). Furthermore, colistin minimum inhibitory concentrations (MICs) >2 µg/mL have been linked to poor prognosis (11). The Clinical and Laboratory Standards Institute (CLSI) has therefore decided to discontinue reporting colistin susceptibility. In line with this, the Infectious Diseases Society of America (IDSA) has downgraded the recommendation for polymyxin use in treating CRAB and other resistant infections (5, 9). Even when polymyxin is used, polymyxin B, which has not been introduced in South Korea, can be considered for moderate to severe CRAB infections (5).

The IDSA 2022 guidance on the treatment of antimicrobial-resistant gram-negative infections started to suggest high-dose ampicillin/sulbactam combined with other susceptible antimicrobial agents as the preferred regimen for moderate to severe CRAB infections, rather than polymyxin-based therapy (12). Prolonged infusion of high-dose ampicillin/sulbactam (sulbactam 9 g/day) may be effective in treating moderate to severe CRAB infections, with sulbactam acting both as a substrate for OXA-carbapenemase and by saturating altered penicillin-binding proteins (PBPs) (5). Meta-analyses have shown that high-dose ampicillin/sulbactam-based regimens for moderate to severe CRAB infections are associated with higher clinical cure and survival rates, as well as fewer side effects, including nephrotoxicity, compared to other regimens (13, 14). However, there is a lack of well-designed, comparative studies evaluating colistin-based combination therapy versus prolonged infusion of high-dose ampicillin/sulbactam-based combination therapy for CRAB pneumonia. Therefore, we conducted a retrospective cohort study to compare the effectiveness and safety of high-dose ampicillin/sulbactam-based combination therapy and colistin-based combination therapy in patients with CRAB nosocomial pneumonia.

## MATERIALS AND METHODS

### Study design and patients

A retrospective cohort study was conducted on adult patients diagnosed with nosocomial pneumonia—either clinically defined (PNU1) or with specific laboratory findings (PNU2) according to the CDC/NHSN surveillance definition—caused by CRAB at Hallym University Chuncheon Sacred Heart Hospital, a university-affiliated hospital in South Korea (15). We enrolled patients who received colistin-based combination therapy with a loading dose followed by a maintenance dose (June 2021 to May 2022) and those who received prolonged infusion (over 4 hours) of high-dose ampicillin/sulbactam-based combination therapy with sulbactam 9 g/day (October 2022 to September 2023).

To be included in the study, patients had to meet the following criteria: (i) age 18 years or older; (ii) repeated isolation of *A. baumannii* from high-quality sputum samples; (iii) *A. baumannii* with a meropenem MIC ≥16 µg/mL; (iv) a diagnosis of ventilator-associated pneumonia or hospital-acquired pneumonia (HAP) as defined by PNU1 or PNU2, and (v) a minimum of 7 days of combination antibiotic therapy for CRAB pneumonia. Patients were excluded if they (i) had not received a colistin loading dose, (ii) received concomitant intravenous and inhaled colistin therapy, (iii) were pregnant, (iv) had a history of allergy to colistin, penicillin, or tetracycline, or (v) were deemed inappropriate for enrollment due to terminal cancer or other severe medical conditions. To ensure accurate identification of true infections and minimize the inclusion of colonization cases, two infectious disease physicians independently assessed each case of CRAB nosocomial pneumonia.

The antibiotic regimens and dosages in this study were determined according to the hospital’s guidelines. Based on their treatment course, patients with CRAB pneumonia were classified into one of two groups. The test group received combination therapy with prolonged infusion (over 4 hours) of high-dose ampicillin/sulbactam (sulbactam 9 g IV every 8 hours), along with oral minocycline (200 mg loading dose followed by 100 mg–200 mg twice daily as maintenance therapy) from October 2022 to September 2023 (5). In the active concurrent control group, patients received intravenous colistin (loading dose followed by maintenance doses) combined with another antibiotic from June 2021 to May 2022 (8, 9). Dosage adjustments for all patients were carefully made according to their individual renal function. Patients were followed for 28 days after treatment initiation to assess clinical outcomes.

### Data collection

Patient demographics (age, gender, Charlson Comorbidity Index [CCI], underlying medical conditions, recent surgical history), baseline status at treatment initiation for CRAB pneumonia (temperature, consciousness, need for vasopressors or mechanical ventilation, Sequential Organ Failure Assessment (SOFA) score, serum creatinine, serum albumin, white blood cell count), and details about the infections (such as whether ventilator-associated pneumonia was present, the MIC distribution for meropenem, colistin, and ampicillin/sulbactam in CRAB, and the presence of CRAB bacteremia) were collected. The use of corticosteroids was defined as ≥20 mg of prednisolone or equivalent over 3 weeks or ≥10 mg/day for at least 3 months. Information on the regimen and duration of combination therapy was also gathered. Species identification and antibiotic susceptibility tests for CRAB isolates were performed using the VITEK 2 system (bioMerieux, Marcy I’Etoile, France) according to the Clinical and Laboratory Standards Institute M100S guidelines (16). *A. baumannii* isolates with an MIC >4 µg/mL for imipenem and meropenem were classified as carbapenem-resistant. Isolates with a colistin MIC ≤2 µg/mL were classified as susceptible until May 2022. Since June 2022, CLSI no longer reported susceptibility to polymyxins for *A. baumannii* and has established an MIC of ≤2 µg/mL as intermediate and an MIC of ≥4 µg/mL as resistant to colistin. *A. baumannii* isolates with an ampicillin/sulbactam MIC of ≤8 µg/mL were reported as susceptible, 16 µg/mL as intermediate, and ≥32 µg/mL as resistant. In VITEK 2 version 8.01, which was used until May 2022, the detection of a carbapenemase phenotype in *A. baumannii* resulted in the susceptibility interpretation for ampicillin/sulbactam being classified as resistant, even if the MIC values indicated susceptibility or intermediate resistance. However, starting in June 2022, with the implementation of version 9.02, the susceptibility interpretation for ampicillin/sulbactam was no longer adjusted based on carbapenemase phenotype detection. Instead, the MIC values were reported directly as susceptible, intermediate, or resistant.

### Outcome measurement

The primary outcome was in-hospital 28-day all-cause mortality following treatment initiation for CRAB nosocomial pneumonia with either colistin-based or high-dose ampicillin/sulbactam-based combination therapy. In-hospital mortality was defined as death occurring during the same hospital admission. Secondary outcomes included clinical success and kidney injury scores based on the Risk, Injury, Failure, Loss, End-stage renal disease (RIFLE) criteria at 14 and 28 days after treatment initiation (17–19). Clinical success was a composite outcome consisting of five elements: (i) survival, (ii) hemodynamic stability (systolic blood pressure >90 mmHg without the need for vasopressor support), (iii) stable or improved SOFA score (for baseline SOFA ≥3, a decrease of at least 30%, and for baseline SOFA <3, stable or decreased score), (iv) stable or improved respiratory status (PaO_2_/FiO_2_), and (v) no growth of CRAB in blood cultures taken from patients with pneumonia and bacteremia who remained febrile. Failure to meet any of these five criteria was defined as clinical failure (17, 18). Additional outcomes included microbiologic failure (identification of CRAB with the same antimicrobial susceptibility pattern in respiratory specimens after at least 7 days of treatment), the presence of superinfections by day 28, and the occurrence of colistin-resistant organisms or *Clostridioides difficile* infections by day 28.

### Statistical analysis

The null hypothesis is that there is no difference in treatment outcomes between the two groups, while the alternative hypothesis is that the effectiveness and safety of high-dose ampicillin/sulbactam-based combination therapy are superior to those of colistin-based combination therapy. To test this hypothesis, the sample size was calculated based on the expected incidence of the primary outcome, 28-day all-cause mortality, as reported in previous studies: 0.09 in the high-dose ampicillin/sulbactam group and 0.26 in the colistin group (14). Using a significance level of 0.05 and a statistical power of 0.8, we determined that at least 83 patients per group would be required to detect a significant difference between the two groups. To account for a potential 10% unassessable rate, the target enrollment was set at approximately 182 participants.

The primary outcome (28-day all-cause mortality) and secondary outcomes (14-day/28-day clinical success rates and 14-day/28-day kidney injury using the RIFLE score) were compared between the two groups using a combination of logistic regression and Poisson regression, depending on the type of outcome variable. Logistic regression was applied to binary outcomes, such as 28-day all-cause mortality and clinical cure at 14 and 28 days, to estimate adjusted relative risks (RRs), corresponding 95% confidence intervals (CIs), and *P*-values. Poisson regression was used for the RIFLE-based kidney injury scores at 14 and 28 days to estimate adjusted RRs, CIs, and *P*-values. Wald tests were used to derive *P*-values for the significance of coefficients in both regression models. Missing data in the RIFLE-based kidney injury variables were imputed using the multiple imputation method, specifically through an iterative imputer in Python, which performs regression modeling on incomplete data using other available variables to predict and impute missing values. Additionally, Cox proportional hazards regression was conducted to identify confounding factors that might independently contribute to the primary outcome (28-day all-cause mortality) beyond the two treatment regimens. Kaplan-Meier analysis with a log-rank test was also performed to compare 28-day survival following treatment initiation for CRAB nosocomial pneumonia (time-to-event outcome) between the two groups. All statistical analyses were performed using R version 4.3.2 and Python version 3.8.

## RESULTS

### Flowchart of patient enrollment and characteristics of the enrolled patients

**Fig 1:**
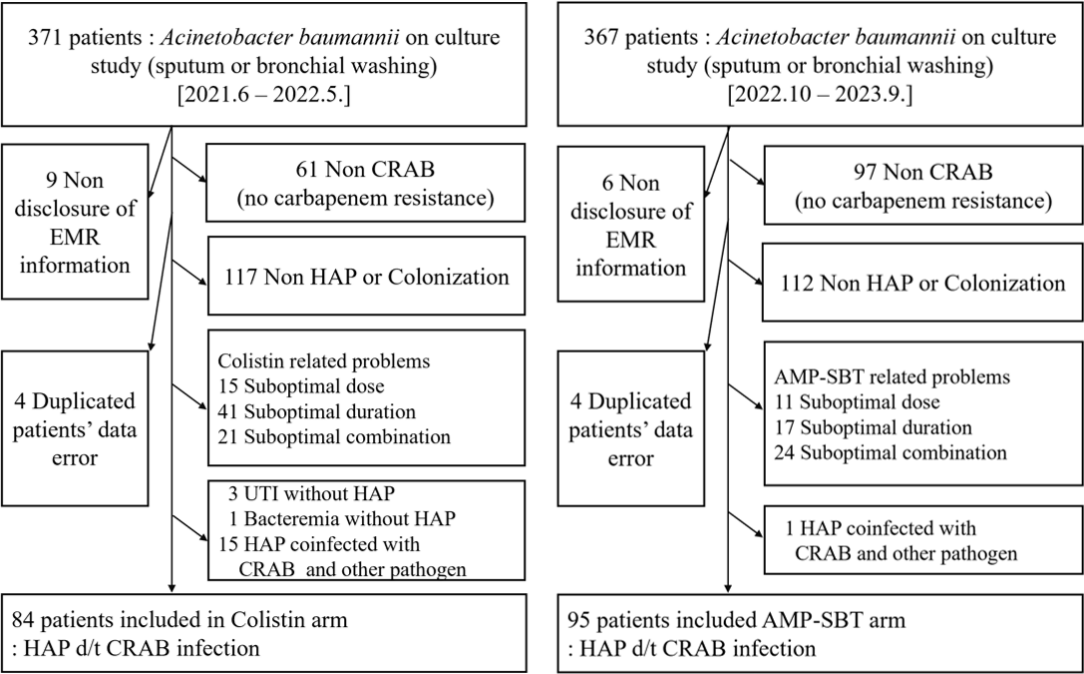
Flowchart of patient enrollment based on study screening criteria.

A total of 738 patients were screened for CRAB nosocomial pneumonia and treated with either colistin-based combination therapy (June 2021 to May 2022) or high-dose ampicillin/sulbactam-based combination therapy (October 2022 to September 2023). Ultimately, 84 out of 371 patients were enrolled in the colistin group, and 95 out of 367 patients were enrolled in the high-dose ampicillin/sulbactam group (Fig. 1).

The baseline characteristics of patients with CRAB nosocomial pneumonia were similar between the colistin and high-dose ampicillin/sulbactam groups, with some notable differences (Table 1). The median age of the enrolled patients was 74 years, and 122 (68%) were male. There was no significant difference in the CCI (4.3 vs 4.2) or SOFA scores (3.1 vs 2.9) between the two groups at the start of treatment for CRAB pneumonia. Baseline serum creatinine levels (1.0 vs 0.9) and the need for vasopressors (11% vs 8%) were also comparable. Rates of ventilator-associated pneumonia (54% vs 57%) and CRAB bloodstream infections with pneumonia as the primary source (13% vs 16%) were similar. Although the number of patients undergoing chemotherapy within 3 months was small, a higher percentage was observed in the colistin group (11% vs 1%).

**TABLE 1:** Characteristics of enrolled patients^*b*^

Characteristic	Colistin arm (*n* = 84)	Ampicillin/sulbactam arm (*n* = 95)	*P*-value^*a*^
Baseline characteristics			
Age (years)	75 (67–84)	73 (66–83)	0.159
Sex, male	60 (71)	62 (65)	0.423
CCI	4.3 (3.0–5.0)	4.2 (3.0–5.0)	0.652
PNU1	36 (43)	42 (44)	0.881
PNU2	48 (57)	53 (56)	0.881
SOFA score	3.1 (1.0–4.0)	2.9 (2.0–4.0)	0.944
Ventilator-associated pneumonia	45 (54)	54 (57)	0.763
Pneumonia with CRAB bacteremia	11 (13)	15 (16)	0.674
Need for vasopressor	9 (11)	8 (8)	0.620
Serum creatinine level	1.0 (0.5–1.1)	0.9 (0.5–1.0)	0.899
Serum albumin level	3.0 (2.8–3.2)	3.4 (2.9–3.3)	0.104
Underlying diseases or conditions			
Diabetes mellitus	38 (45)	41 (43)	0.880
Hypertension	46 (55)	47 (49)	0.549
Congestive heart failure	7 (8)	14 (15)	0.245
Chemotherapy within 3 months	9 (11)	1 (1)	**0.007**
Corticosteroid use within 3 months	3 (4)	7 (7)	0.339
Surgery within 3 months	18 (21)	26 (27)	0.388
MIC distribution of antibiotics for CRAB isolates			
Meropenem (≥16 µg/mL)	84 (100)	95 (100)	1.000
Colistin (<0.5 µg/mL)	83 (99)	94 (99)	0.999
(1 µg/mL)	1 (1)	1 (1)	0.999
Ampicillin/sulbactam			
(≥32 µg/mL)	35 (42)	84 (88)	**<0.001**
(16 µg/mL)	44 (52)	10 (11)	**<0.001**
(8 µg/mL)	4 (5)	1 (1)	0.188
(4 µg/mL)	1 (1)	0 (0)	0.469
Combination regimen for CRAB			
Meropenem or imipenem	39 (43)		
Cefepime	22 (24)		
Piperacillin/tazobactam ± (levofloxacin or minocycline)	14 (15)		
Ceftazidime or cefotaxime	8 (9)		
Minocycline	6 (7)	95 (100)	
Levofloxacin or ciprofloxacin	2 (2)		
Duration of combination therapy	12.6 (7.0–14.0)	12.5 (8.0–15.0)	0.359
Clinical outcome			
28-day mortality	51 (61)	19 (20)	**<0.001**
14-day clinical cure	19 (23)	37 (39)	**0.024**
28-day clinical cure	27 (32)	52 (55)	**0.003**
14-day RIFLE score-based kidney injury	0.88 (0.0–1.0)	0.77 (0.0–1.0)	**0.034**
28-day RIFLE score-based kidney injury	1.06 (1.0–1.06)	0.63 (0.0–0.63)	**<0.001**
Infection-related clinical events			
Microbiologic failure	49 (58)	33 (35)	**0.002**
Superinfection within 28 days	22 (26)	23 (24)	0.863
Emergence of colistin-resistant organisms within 28 days	2 (2)	2 (2)	1.000
*Clostridioides difficile* infections	3 (4)	4 (4)	1.000

^*a*^ Continuous variables were compared using the Mann-Whitney U-test, and categorical data were compared using Fisher’s exact test. Bold font indicates *P* < 0.05.

^*b*^ Note: Data are presented as median (interquartile range) or *n* (%).

All CRAB isolates in this study exhibited meropenem MIC ≥16 µg/mL, colistin MIC ≤1 µg/mL, and ampicillin/sulbactam MIC ≥4 ug/mL. Notably, in 99% of enrolled patients, colistin MICs were ≤0.5 µg/mL, and 97% had ampicillin/sulbactam MICs ≥16 µg/mL. In the colistin group tested with VITEK 2 version 8.01, 42 of the 44 CRAB isolates with an ampicillin/sulbactam MIC of 16 µg/mL were reported as resistant to ampicillin/sulbactam. Additionally, all five CRAB isolates with an ampicillin/sulbactam MIC of 4–8 µg/mL were also found to be resistant to ampicillin/sulbactam (Table 1). Despite observed resistance, colistin-based regimens frequently included carbapenems (43%), cefepime (24%), or piperacillin/tazobactam (15%). In contrast, all high-dose ampicillin/sulbactam-based regimens included minocycline, even in cases where minocycline resistance was present (3%). The duration of combination therapy was similar between the two groups (12.6 vs 12.5 days).

### Comparisons of clinical outcomes between colistin-based and high-dose ampicillin/sulbactam-based therapies in patients with CRAB nosocomial pneumonia

In a univariate analysis, the high-dose ampicillin/sulbactam group demonstrated lower 28-day all-cause mortality, higher clinical cure rates at 14 and 28 days, and lower RIFLE score-based kidney injury at both 14 and 28 days. Additionally, microbiologic failure was less frequent in the high-dose ampicillin/sulbactam group (Table 1).

In a multivariable analysis, the 28-day mortality rate in the high-dose ampicillin/sulbactam group (20%) was significantly lower than that in the colistin group (61%) (adjusted RR = 0.16, 95% CI 0.08–0.32; *P* < 0.001). The 14-day clinical success rate was also higher in the high-dose ampicillin/sulbactam group (39%) compared to the colistin group (23%) (aRR = 2.19, 95% CI 1.10–4.37; *P* = 0.026). Similarly, the 28-day clinical success rate was higher in the ampicillin/sulbactam group (55%) than in the colistin group (32%) (aRR = 2.71, 95% CI 1.14–5.20; *P* = 0.003). Additionally, the 28-day RIFLE score-based kidney injury was lower in the high-dose ampicillin/sulbactam group (0.63 ± 1.16) compared to the colistin group (1.06 ± 1.35) (aRR = 0.56, 95% CI 0.40–0.79; *P* = 0.001) (Table 2).

**TABLE 2:** Comparison of clinical outcomes between colistin-based combination therapy and high-dose ampicillin/sulbactam-based combination therapy using multivariable analysis

	Colistin (84)	Ampicillin/sulbactam (95)	RR	95% CI	*P*-value
Primary outcome					
28-day all-cause mortality	51 (61%)	19 (20%)	0.16	0.08–0.32	<0.001
Secondary outcome					
Clinical cure at 14 days	19 (23%)	37 (39%)	2.19	1.10–4.37	0.026
Clinical cure at 28 days	27 (32%)	52 (55%)	2.71	1.14–5.20	0.003
RIFLE score-based kidney injury at 14 days (mean ± SD)	0.88 ± 1.17	0.77 ± 1.25	0.85	0.60–1.19	0.337
RIFLE score-based kidney injury at 28 days (mean ± SD)	1.06 ± 1.35	0.63 ± 1.16	0.56	0.40–0.79	0.001

### Risk factors for 28-day all-cause mortality and differences in 28-day mortality between treatment groups in patients with CRAB nosocomial pneumonia

Univariate analysis results for factors associated with 28-day all-cause mortality are presented in Table 3. In addition to the colistin-based combination regimen, higher SOFA scores (3.5 vs 2.6), higher CCI scores (4.7 vs 4.0), use of cytotoxic chemotherapy within 3 months (11% vs 2%), lower clinical cure rates at 14 and 28 days (9% vs 46% and 11% vs 65%, respectively), higher RIFLE score-based kidney injury at 14 and 28 days (1.0 vs 0.7 and 1.0 vs 0.8), and higher rates of microbiologic failure (76% vs 27%) were associated with increased mortality. Conversely, factors associated with 28-day survival, aside from high-dose ampicillin/sulbactam-based combination therapy, included recent surgery within the past 3 months (16% vs 30%).

**TABLE 3:** Characteristics of patients and univariate analysis of factors associated with all-cause mortality within 28 days^*b*^

Characteristic	Mortality within 28 days (*n* = 70)	Survival within 28 days (*n* = 109)	*P*-value^*a*^
Baseline characteristics			
Age (years)	76 (72–84)	72 (65–84)	0.070
Gender, male	47 (67)	75 (69)	0.870
CCI	4.7 (4.0–6.0)	4.0 (3.0–5.0)	**0.027**
PNU1	33 (47)	45 (41)	0.445
PNU2	37 (53)	64 (59)	0.445
SOFA score	3.5 (2.0–5.0)	2.6 (1.0–3.0)	**0.017**
Ventilator-associated pneumonia	42 (60)	57 (52)	0.357
Pneumonia with CRAB bacteremia	14 (20)	12 (11)	0.128
Need for vasopressor	9 (13)	8 (7)	0.296
Serum creatinine level	1.0 (0.5–1.1)	1.0 (0.5–1.0)	0.960
Underlying diseases or conditions			
Diabetes mellitus	35 (50)	44 (40)	0.220
Hypertension	33 (47)	60 (55)	0.358
Congestive heart failure	6 (9)	15 (14)	0.348
Chemotherapy within 3 months	8 (11)	2 (2)	**0.015**
Corticosteroid use within 3 months	4 (6)	6 (6)	1.000
Surgery within 3 months	11 (16)	33 (30)	**0.033**
MIC distribution of antibiotics for CRAB isolates			
Meropenem (≥16 µg/mL)	70 (100)	109 (100)	1.000
Colistin (<0.5 µg/mL)	70 (100)	107 (98)	0.521
(1 µg/mL)	0 (0)	2 (2)	0.521
Ampicillin/sulbactam			
(≥32 µg/mL)	39 (56)	80 (73)	**0.016**
(16 µg/mL)	28 (40)	26 (24)	**0.030**
(8 µg/mL)	3 (4)	2 (2)	0.381
(4 µg/mL)	0 (0)	1 (1)	1.000
Combination therapy regimen			
Colistin-based regimen	51 (73)	33 (30)	**<0.001**
High-dose ampicillin/sulbactam regimen	19 (27)	76 (70)	**<0.001**
Duration of combination therapy	11.84 (7.0–14.0)	12.31 (8.0–15.0)	0.376
Clinical outcome			
14-day clinical cure	6 (9)	50 (46)	**<0.001**
28-day clinical cure	8 (11)	71 (65)	**<0.001**
14-day RIFLE score-based kidney injury	1.00 (0.77–1.0)	0.70 (0.0–1.0)	**<0.001**
28-day RIFLE score-based kidney injury	0.96 (0.63–1.06)	0.75 (0.0–1.0)	**<0.001**
Infection-related clinical events			
Microbiologic failure	53 (76)	29 (27)	**<0.001**
Superinfection within 28 days	20 (29)	25 (23)	0.480
Emergence of colistin-resistant organisms within 28 days	2 (3)	2 (2)	0.645
*Clostridioides difficile* infection	1 (1)	6 (5)	0.249

^*a*^ Continuous variables were compared using the Mann-Whitney U-test, and categorical data were compared using Fisher’s exact test. Bold font indicates *P* < 0.05.

^*b*^ Note: Data are presented as median (interquartile range) or *n* (%).

**Fig 2:**
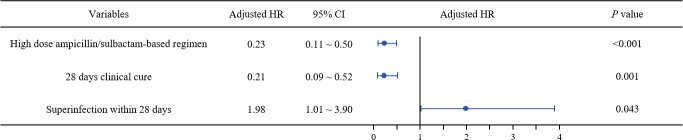
Risk factors for all-cause mortality within 28 days in patients with CRAB nosocomial pneumonia identified using Cox proportional hazards regression.

In Cox proportional hazards regression analysis, high-dose ampicillin/sulbactam-based combination therapy (adjusted hazard ratio [aHR] = 0.23, 95% CI 0.11–0.50; *P* < 0.001), and achieving a 28-day clinical cure (aHR = 0.21, 95% CI 0.09–0.52; *P* = 0.001) were each significantly associated with reduced all-cause mortality within 28 days. In contrast, superinfection within 28 days (aHR = 1.98, 95% CI 1.01–3.90; *P* = 0.048) was associated with increased 28-day all-cause mortality (Fig. 2; Table S1).

**Fig 3:**
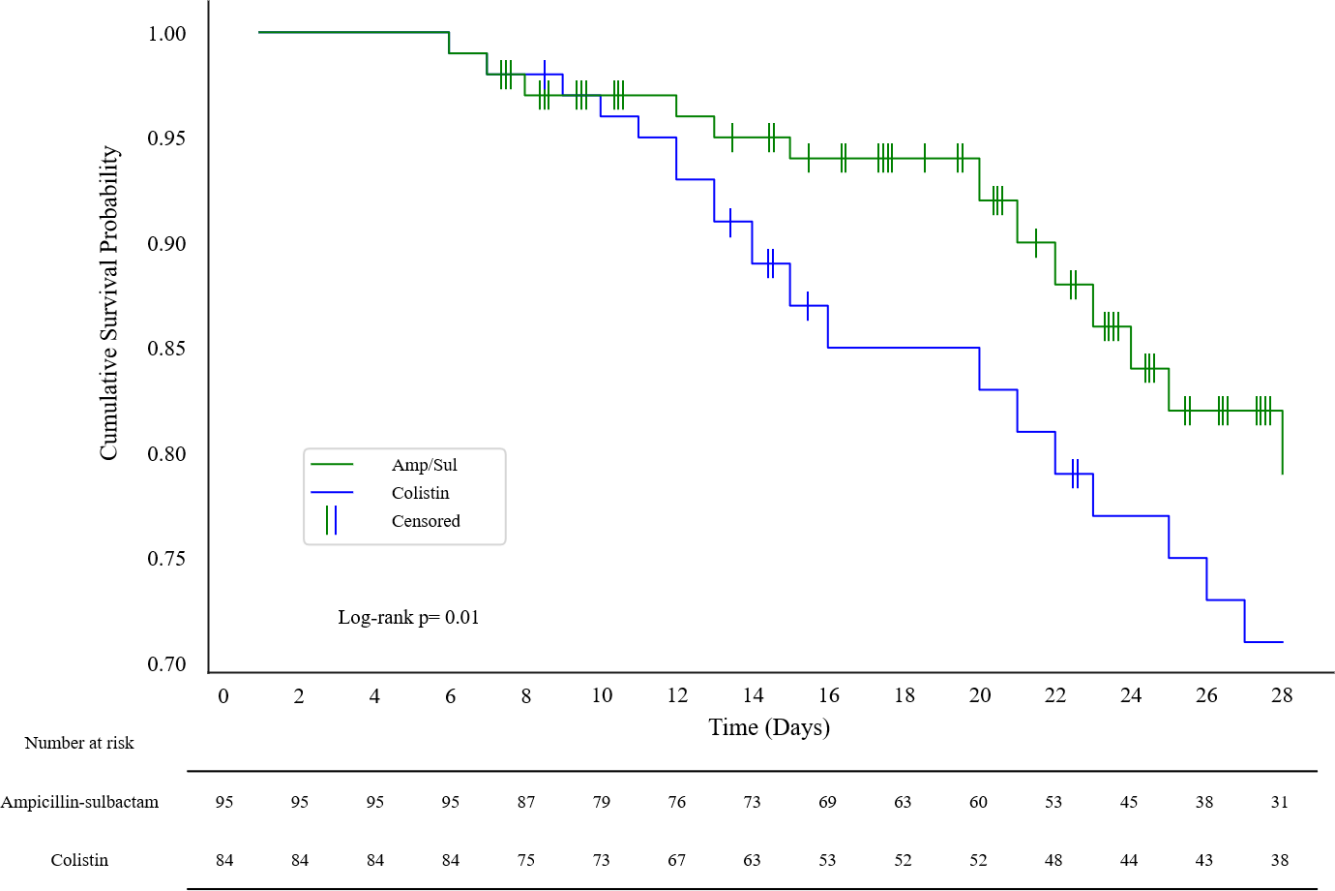
Kaplan-Meier survival analysis of all-cause mortality within 28 days in patients with CRAB nosocomial pneumonia receiving colistin-based combination therapy versus high-dose ampicillin/sulbactam-based combination therapy.

In Kaplan-Meier survival analysis, the cumulative survival probability at 28 days in the high-dose ampicillin/sulbactam-based combination therapy group (0.79) was significantly higher than in the colistin-based combination therapy group (0.71) (log-rank *P* = 0.01) (Fig. 3).

## DISCUSSION

In this study, we compared treatment outcomes of high-dose ampicillin/sulbactam-based combination therapy with those of colistin-based combination therapy for CRAB pneumonia. Multivariable analysis of clinical outcomes showed a significantly lower 28-day all-cause mortality rate in the high-dose ampicillin/sulbactam group (20%) compared to the colistin group (61%). Additionally, the high-dose ampicillin/sulbactam group demonstrated higher clinical success rates at both 14 days (39% vs 23%) and 28 days (55% vs 32%), along with a lower 28-day RIFLE score-based kidney injury (0.63 ± 1.16 vs 1.06 ± 1.35). Cox proportional hazards regression analysis confirmed a significant association between high-dose ampicillin/sulbactam therapy and reduced all-cause mortality within 28 days. Moreover, Kaplan-Meier analysis showed a significantly higher 28-day cumulative survival probability in the high-dose ampicillin/sulbactam group (0.79) than in the colistin group (0.71).

According to the 2024 IDSA guidance for CRAB infection treatment, the preferred regimen is sulbactam-durlobactam combined with a carbapenem. When sulbactam-durlobactam is unavailable, high-dose ampicillin/sulbactam combined with at least one other agent, such as polymyxin B, minocycline, or cefiderocol, is recommended as an alternative (5, 20). The 2022 ESCMID guidelines for moderate to severe CRAB infections suggest combination therapy with two *in vitro* active agents from available antibiotics (polymyxin, aminoglycoside, tigecycline, and sulbactam combinations), with a preference for polymyxin- or high-dose tigecycline-based regimens (7). In settings where newer agents like sulbactam-durlobactam and cefiderocol are unavailable, and colistin is commonly used instead of polymyxin B, this study’s comparison of high-dose ampicillin/sulbactam-based combination therapy with colistin-based therapy for CRAB nosocomial pneumonia is particularly relevant. Although this is a single-center, retrospective cohort study rather than a rigorously controlled randomized controlled trial (RCT), it is notable for its calculated sample size, strict inclusion and exclusion criteria, and minimal differences in baseline characteristics between groups. The findings suggest that prolonged infusion of high-dose ampicillin/sulbactam-based combination therapy is a better way to treat patients with CRAB nosocomial pneumonia, as it offers lower mortality, higher clinical success rates, and reduced kidney injury risk compared to colistin-based combination therapy.

This study focused on CRAB pneumonia cases with isolates exhibiting meropenem MICs of ≥16 µg/mL, colistin MICs of ≤0.5 µg/mL, and ampicillin/sulbactam MICs of ≥16 µg/mL. As polymyxin B is unavailable in South Korea, we compared outcomes between colistin-based combination therapy—administered with loading and maintenance doses adjusted for renal function—and high-dose ampicillin/sulbactam-based combination therapy (8, 9).

Among the antibiotics considered for combination with colistin, aminoglycosides posed a high risk of nephrotoxicity. Although the 2022 ESCMID guidelines recommended high-dose tigecycline as the preferred tetracycline derivative for CRAB infections, a retrospective study of patients with CRAB bacteremia, where pneumonia accounted for 64% of the infection sources, found no difference in 30-day overall mortality between colistin-tigecycline combination therapy and colistin monotherapy (21). Additionally, the use of high-dose tigecycline in South Korea remains challenging due to the lack of insurance coverage.

Clinical data on the combination of colistin and minocycline are limited. In a multicenter retrospective study of various colistin-based combination therapies for CRAB infections, including pneumonia, the combination of colistin and minocycline demonstrated a significantly higher microbiologic response by day 14. However, it was not superior to other combination therapies in terms of 28-day mortality or clinical improvement at days 14 and 28 (22). Ideally, we would have directly compared the clinical outcomes of colistin and high-dose ampicillin/sulbactam therapies combined with minocycline. In this study, minocycline was used in all cases within the high-dose ampicillin/sulbactam group, whereas the colistin group primarily combined colistin with carbapenem, cefepime, and piperacillin/tazobactam, which are *in vitro* inactive agents (Table 1). This was likely due to the relatively low minocycline susceptibility in the colistin group (46%), limiting its use in combination regimens. A study evaluating the *in vitro* synergy of antimicrobial agents in combinations with colistin against colistin-resistant *Acinetobacter baumannii* found potential benefits with rifampin, glycopeptides, or β-lactams (e.g., aztreonam, ceftazidime, meropenem), while combinations with ampicillin-sulbactam, tigecycline, amikacin, azithromycin, or trimethoprim-sulfamethoxazole showed limited synergy (23). Based on these findings, the synergistic effect of colistin combined with minocycline is likely to be limited. However, further studies directly comparing the outcomes of colistin versus high-dose ampicillin/sulbactam combined with minocycline may be needed.

In the high-dose ampicillin/sulbactam group, dosing was based on PK studies and Monte Carlo simulations for sulbactam, which indicate that a daily dose of sulbactam 9 g achieves a high probability of target attainment (over 90%) with 60% T > MIC for *A. baumannii* isolates with a sulbactam MIC of 16 µg/mL (24). Prior studies showing that sulbactam concentrations in the pulmonary epithelial lining fluid reach 61% of serum levels support the use of a sulbactam 9 g daily dose over lower doses for CRAB pneumonia treatment (25). This dosing approach was consistent with the sulbactam dose used in our high-dose ampicillin/sulbactam group. OXA carbapenemases in CRAB—particularly OXA-23 and OXA-51, which are highly prevalent in South Korea—make β-lactams, including carbapenems and sulbactam, largely ineffective (5, 26, 27). Sulbactam resistance is driven by β-lactamases and PBP mutations (PBP1a/1b, PBP3). While ampicillin/sulbactam lacks a β-lactamase inhibitor like durlobactam and is extensively hydrolyzed, high-dose ampicillin-sulbactam is often favored due to its potential to saturate sulbactam’s PBP targets (5). However, the effectiveness of high-dose ampicillin/sulbactam against CRAB isolates producing sulbactam-hydrolyzing enzymes—such as TEM-1 β-lactamase, ACC-30, and Ambler class B metallo-β-lactamase, which are found in clinical CRAB isolates—were not assessed in this study (28, 29). Given this uncertainty, it is advisable to combine high-dose ampicillin/sulbactam with antibiotics that have confirmed *in vitro* activity against CRAB (5). Since agents like polymyxin B and cefiderocol are unavailable in South Korea, minocycline combination therapy was used in the high-dose ampicillin/sulbactam group in this study. This included three cases (3%) of CRAB pneumonia with minocycline resistance (MIC ≥8 µg/mL) and two cases with MICs of 2 µg/mL–4 µg/mL, where susceptibility was reported but suboptimal antibiotic concentrations might be present at sites of infection (30, 31).

There are several limitations in this study. First, following the 2022 IDSA treatment guidance that suggests high-dose ampicillin/sulbactam as a preferred therapy for moderate to severe CRAB infections, our hospital updated its treatment protocol. As a result, it became necessary to evaluate whether there were differences in treatment outcomes between regimens before and after this change (12). However, the different study periods (colistin group: June 2021 to May 2022; high-dose ampicillin/sulbactam group: October 2022 to September 2023) may have influenced the observed differences in treatment results, despite calculated sample size, strict inclusion and exclusion criteria, and minimal differences in baseline characteristics between groups. Second, the combination treatment regimen was not standardized between the two groups. We did not directly compare the clinical outcomes of colistin and high-dose ampicillin/sulbactam therapies combined with minocycline. Clinical data on the combination of colistin and minocycline are limited. Based on studies evaluating the *in vitro* synergy of antimicrobial agents combined with colistin against *Acinetobacter baumannii*, the synergistic effect of colistin with minocycline is likely limited (23, 32). In real-world practice, clinicians at our hospital rarely used the colistin and minocycline combination therapy for treating CRAB pneumonia. Furthermore, the relatively low minocycline susceptibility (46%) of CRAB isolates in the colistin group limited the use of minocycline in combination therapy. In contrast, all patients in the high-dose ampicillin/sulbactam group, where minocycline susceptibility was high (97%), received combination therapy with minocycline, as it was the only available agent in South Korea among the combination regimens recommended by the IDSA guidance for treating CRAB infections. However, further studies directly comparing the outcomes of colistin versus high-dose ampicillin/sulbactam combined with minocycline may be needed. Third, there were some imbalances in baseline characteristics between the two groups. Although the numbers were small, the colistin group included more patients who had received chemotherapy within 3 months. Patients for both treatment groups were selected during the study period based on the enrollment flowchart, ensuring a sample size adequate for statistical power. While propensity score matching could have been used to balance baseline characteristics, this would have resulted in an insufficient sample size for comparing treatment outcomes. Therefore, multivariable analysis was performed to adjust for these baseline differences. Fourth, we limited the patient population to those receiving antibiotic treatment for more than 1 week for CRAB nosocomial pneumonia, as we believed that including patients who died early due to deterioration in their overall condition could confound the evaluation of treatment outcomes between the two groups. Fifth, better treatment options for CRAB are now available, with sulbactam-durlobactam plus a carbapenem as the preferred regimen (5). The sulbactam-durlobactam and carbapenem combination lowers sulbactam-durlobactam’s MIC by one- to twofold (33). Durlobactam enhances the targeting of PBPs by both sulbactam and the carbapenem, with the carbapenem acting as a substrate for OXA-carbapenemase, facilitating sulbactam delivery. However, to reduce selective pressure for carbapenem resistance, early discontinuation of the carbapenem after clinical improvement may be warranted. Cefiderocol, as part of a combination regimen, is primarily reserved for treating CRAB infections that are refractory to other antibiotics or when intolerance or resistance limits the use of alternative agents, given its *in vitro* activity against carbapenem-resistant Enterobacterales or carbapenem-resistant *Pseudomonas aeruginosa* producing metallo-β-lactamases (5). Further well-designed studies are needed to compare clinical outcomes between high-dose ampicillin/sulbactam-based combination regimens (with polymyxin B, minocycline, or cefiderocol) and direct comparisons with sulbactam-durlobactam plus a carbapenem.

In conclusion, prolonged infusion of high-dose ampicillin/sulbactam-based combination therapy is a better way to treat patients with CRAB nosocomial pneumonia, as it is associated with lower mortality, a higher clinical success rate, and a reduced risk of kidney injury compared to colistin-based combination therapy.

## SUPPLEMENTAL MATERIAL

Table S1 aac.01880-24-s0001.docx Multivariable Cox proportional hazards model for 28-day all-cause mortality.

ASM does not own the copyrights to Supplemental Material that may be linked to, or accessed through, an article. The authors have granted ASM a non-exclusive, world-wide license to publish the Supplemental Material files. Please contact the corresponding author directly for reuse.
