# Supplementary material for: Comparative effectiveness and safety of colistin-based versus high-dose ampicillin/sulbactam-based combination therapy for nosocomial pneumonia caused by carbapenem-resistant Acinetobacter baumannii
Source: Antimicrob Agents Chemother. 2025 Apr 23;69(6):e01880-24. doi: 10.1128/aac.01880-24 (PMC12135504; doi:10.1128/aac.01880-24)
Supplement: Table S1 — Multivariable Cox proportional hazards model for 28-day all-cause mortality. [file aac.01880-24-s0001.docx]

**Supplemental Table 1. Multivariable Cox Proportional Hazards Model for 28-Day All-Cause Mortality.**

| Covariate | HR | 95% CI | P-Value |
| --- | --- | --- | --- |
| **High dose Ampicillin/Sulbactam-based regimen** | 0.23 | 0.11 ~ 0.50 | <0.001 |
| **28-day clinical cure** | 0.21 | 0.09 ~ 0.52 | 0.001 |
| **Superinfection within 28 days** | 1.98 | 1.01 ~ 3.90 | 0.048 |
| Microbiologic failure | 1.99 | 0.99 ~ 3.99 | 0.053 |
| 14-day clinical cure | 0.41 | 0.15 ~ 1.09 | 0.074 |
| Pneumonia with CRAB bacteremia | 1.69 | 0.83 ~ 3.44 | 0.146 |
| MIC for Minocycline | 0.67 | 0.38 ~ 1.17 | 0.158 |
| SOFA score | 1.10 | 0.96 ~ 1.27 | 0.159 |
| Diabetes mellitus | 1.56 | 0.84 ~ 2.91 | 0.161 |
| Hypertension | 0.67 | 0.36 ~ 1.22 | 0.185 |
| Serum creatinine level | 0.79 | 0.52 ~ 1.19 | 0.250 |
| *Clostridioides difficile* infections | 0.27 | 0.03 ~ 2.51 | 0.251 |
| Gender | 1.40 | 0.77 ~ 2.54 | 0.268 |
| Surgery within 3 months | 0.76 | 0.34 ~ 1.70 | 0.502 |
| steroid therapy within 3 months | 1.47 | 0.46 ~ 4.66 | 0.514 |
| Pneumonia type according to the CDC/NHSN surveillance definition | 1.18 | 0.68 ~ 2.06 | 0.550 |
| Emergence of colistin-resistant organisms | 0.63 | 0.12 ~ 3.29 | 0.586 |
| MIC for Ampicillin/Sulbactam | 1.14 | 0.67 ~ 1.96 | 0.625 |
| chemotherapy within 3 months | 1.32 | 0.43 ~ 4.03 | 0.626 |
| Age | 1.01 | 0.98 ~ 1.04 | 0.685 |
| Serum albumin level | 0.98 | 0.80 ~ 1.20 | 0.821 |
| Need for ventilator | 1.07 | 0.58 ~ 1.95 | 0.829 |
| ICU admission status | 0.90 | 0.34 ~ 2.37 | 0.835 |
| 14-day RIFLE score-based kidney injury | 1.03 | 0.76 ~ 1.41 | 0.839 |
| Duration of combination therapy | 0.99 | 0.94 ~ 1.05 | 0.842 |
| Congestive heart failure | 0.91 | 0.30 ~ 2.73 | 0.862 |
| 28-day RIFLE score-based kidney injury | 1.04 | 0.68 ~ 1.59 | 0.862 |
| Need for vasopressor | 1.05 | 0.43 ~ 2.56 | 0.916 |
| Charlson Comorbidity Index | 0.99 | 0.79 ~ 1.25 | 0.948 |
| MIC for Colistin | 0.00 | 0.00 ~ inf | 0.996 |
